# Targeted sequencing to identify genetic alterations and prognostic markers in pediatric T-cell acute lymphoblastic leukemia

**DOI:** 10.1038/s41598-020-80613-6

**Published:** 2021-01-12

**Authors:** Ya-Hsuan Chang, Chih-Hsiang Yu, Shiann-Tarng Jou, Chien-Yu Lin, Kai-Hsin Lin, Meng-Yao Lu, Kang-Hsi Wu, Hsiu-Hao Chang, Dong-Tsamn Lin, Shu-Wha Lin, Hsuan-Yu Chen, Yung-Li Yang

**Affiliations:** 1 Institute of Statistical Science Academia Sinica, 128 Academia Road, Section 2, Nankang, Taipei, 11529 Taiwan; 2 Departments of Clinical Laboratory Sciences and Medical Biotechnology, College of Medicine, National Taiwan University, Taipei, Taiwan; 3 Department of Pediatrics, National Taiwan University Hospital, Taipei, Taiwan; 4 Department of Pediatrics, College of Medicine, National Taiwan University, Taipei, Taiwan; 5 Department of Pediatrics, Chung Shan Medical University Hospital and School of Medicine, Chung Shan Medical University, Taichung, Taiwan; 6 Department of Laboratory Medicine, National Taiwan University Hospital, Taipei, Taiwan; 7 Department of Laboratory Medicine, College of Medicine, National Taiwan University, 100, No 7. Chung-Shan South Road, Taipei, Taiwan

**Keywords:** Medical research, Oncology

## Abstract

T-cell acute lymphoblastic leukemia (T-ALL) is caused by the accumulation of multiple genetic alterations. To determine the frequency of common genetic mutations and possible prognostic markers in childhood T-ALL, we performed targeted sequencing of 67 genes across 64 cases treated according to Taiwan Pediatric Oncology Group protocols between January 2002 and December 2015. Together, 302 variants were identified in 60 genes including 233 single nucleotide variants and 69 indels. Sixty-four samples had a median number of six genetic lesions each (range 1–17). Thirteen genes had mutation frequencies > 10%, and 5 were > 20%, with the highest being *NOTCH1* (70.31%). Protocadherins *FAT1* (32.81%) and *FAT3* (17.19%), and the ubiquitin ligase component *FBXW7* (28.13%) had higher mutation frequencies than previously reported. Other mutation frequencies (*PHF6*, *DNM2*, *DNMT3A, CNOT3*, and *WT1)* were within previously reported ranges. Three epigenetic-related genes (*KMT2D*, *DNMT3A*, and *EZH2)* were mutated in our cohort. *JAK-STAT* signaling pathway genes had mutation frequencies of 3–13% and were observed in 23 cases (35.94%). Changes to genes in the ErbB signaling pathway were detected in 20 cases (31.25%). Patients with *NOTCH1*/*FBXW7* mutations and *RAS*/*PTEN* germline exhibited better 5-year overall survival rates.

## Introduction

Around 15% of cases of childhood acute lymphoblastic leukemia (ALL) is T-cell ALL (T-ALL)^1,2^. Clinically, T-ALL is characterized by a high white cell count, a mediastinal mass, and an inferior outcome compared to the B-cell ALL. With improvement in chemotherapy, supportive care, minimal residual disease (MRD) detection, and the possibility of stem cell transplants, outcomes have gradually improved in the last 10 to 20 years^3^. T-ALL can be classified into subgroups according to the gene expression of various transcription factors, including *TAL1*, *TLX*, *HOXA9/10*, *LMO2*, and *NKX2-1*^4–6^. Deletions of the *CDKN2A* locus are present in about 70% of T-ALLs^7^. Candidate gene sequencing identified several genetic mutations or alterations in T-ALL, including *NOTCH1*, *JAK1*, *IL7R*, *ETV6*, *RUNX1*, *BCL11B*, *LEF1*, *PHF6*, and *WT1*^8–18^. Zhang et al. demonstrated the first comprehensive whole genome sequencing of 12 patients with early T-cell precursor ALL, and assessed their findings in another 94 T-ALL patient samples^19^. Liu et al. used integrated genome analysis to investigate T-ALL samples including whole genome sequencing, whole exome sequencing, and RNA-sequencing. The above studies discovered more complex and heterogeneous genetic somatic mutations in T-ALL, which involve numerous transcriptional, signaling, and epigenetic factor pathways in the pathogenesis of this disease^19–22^.

Half of the T-ALLs show an aberrant expression of transcriptional factors, whose specific breaking points result in the aberrant expression of genes that might require the use of whole genome sequencing (WGS) to identify^20^. The other cases of T-ALL might harbor chromosomal rearrangements, some of which might become therapeutic targets^20^. *NOTCH1* mutations were first identified in more than 50% of T-ALL cases^8^, and many subsequent studies have discussed *NOTCH1* and its related pathway as a prognostic marker in T-ALL^23–29^. Current sequencing efforts have identified several pathways of genetic alteration in T-ALL, including transcription factors, signaling pathways, epigenetics, mistranslation, and RNA stability, in addition to *NOTCH1* signaling^19,20,30,31^. However, these alterations have rarely been used as prognostic stratifications in the treatment of patients or they do not confer consistent prognostic significance in different protocols^10,14,16–19,32–35^. Chemotherapy can be tailored according to minimal residual disease (MRD) data in childhood T-ALL, although MRD in T-ALL may not be good enough to predict final outcomes in the same way that B-ALL can be targeted based on *ETV6-RUNX1* or hyperdiploidy^2,36^. Investigators hope to identify genetic markers to predict T-ALL outcomes more precisely^37,38^. Another benefit of sequencing for T-ALL is the identification of possible genetic alterations suitable for targeted therapy, such as a mutant *JAK-STAT* pathway^39^.

In this study, we used targeted next generation amplicon sequencing (NGS-based amplicon sequencing) to sequence multiple genes, which were reported to be involved in T-ALL, and investigated their prognostic impacts for patients with pediatric T-ALL under treatment with Taiwan Pediatric Oncology Group (TPOG) protocols in Taiwan.

## Materials and methods

### Patients and protocols

The diagnosis of T-ALL was based on bone marrow aspiration or peripheral blood and immune-phenotyping with monoclonal antibodies directed to T-lineage-associated antigens. Early T-cell precursor ALL (ETP-ALL) status was diagnosed based upon the criteria proposed by Coustan-Smith et al.^40^. Between January 2002 and December 2015, 64 pediatric patients with T-ALL were enrolled. All were treated according to the TPOG ALL protocols. Risk-directed TPOG protocols use multiple chemotherapeutic agents of different intensities. Patients with T-ALL were assigned to the very high-risk protocol. After 2013, MRD levels were added to the risk assignment for therapy. Events were defined as any relapse, secondary malignancy, or death. The Institutional Review Board of National Taiwan University Hospital approved the study and all of the participants or their guardians provided written, informed consent, in accordance with the Declaration of Helsinki. Details of the protocols are published elsewhere^41,42^. Genomic DNA was extracted from leukemic bone marrow or peripheral blood, as previously described^42^.

### Determination of gene expression by real time quantitative PCR (RT-PCR)

For samples with available total RNA, cDNA was synthesized using Maxima First Strand cDNA Synthesis Kit (Thermo Fisher Scientific, Waltham, MA, USA) and qPCR was performed using SensiFast Probe No-ROX Kit (Meridian Bioscience, Cincinnati, OH, USA) using the StepOnePlus system (Applied Biosystems, Foster City, CA)^4–6^. We determined the expression of *TAL1, TAL2, LYL1, TLX1, TLX3, LMO1, LMO2, NKX2-1,* and *HOXA* oncogenes, using the *YWHAZ* gene as an internal control.

### Amplicon design and sequencing data processing

NuGEN’s Ovation Target Enrichment System (NuGEN Technologies, Inc., San Carlos, California) was used to design probes and hybridize exon regions of 67 targeted genes. In all, 6465 designed probes of 50 base pairs (bps) were used to enrich targeted regions of 556,600 bps. Sample DNA was fragmented, ligated by adaptors and then hybridized with designed probes. After polymerase chain reaction (PCR) amplification for sequencing library enrichment, products were sequenced by Illumina HiSeq 4000 (Illumina, Inc., San Diego, California), with 150 paired-end reads.

Sequencing reads were examined for quality, and for adaptor or primer sequence contamination, by FastQC (v0.11.5). After removing unqualified bases (average quality base < 20 per 4-base sliding window) by Trimmomatic (v0.36), reads were aligned to human reference genome GRC37/hg19 by BWA (v0.7.17). In order to reduce the effect of systematic sequencing errors from sequencers, and the false positive rate in identifying variants, quality score recalibration and marked duplication were both applied to reads by GATK (v4.0). Finally, variants were identified by VarScan (v2.3.9). Variants meeting the following criteria were selected for further analysis: (1) with ≥ 10 reads; (2) ≥ 2 reads with alternative alleles; (3) alternative allele fraction (AF) ≥ 1%; (4) average base quality ≥ 15; (5) without > 90% reads supported by one strand (6) ≥ 7 samples with variants (7) carrier frequency < 1% in East Asian populations of the 1000 Genome Project.

### Statistical analysis

In order to clarify whether genetic profiles of *NOTCH1*, *FBXW7*, *RAS,* and *PTEN* were prognostic markers, as previously reported, variants present in the COSMIC database (v70) were selected and survival analysis was carried out, to compare the difference of time to induction failure or survival between genetic risk groups. The Kaplan–Meier method was used to generate survival curves. The log-rank test was applied to compare the difference between survival curves. Adjusted hazard ratio was estimated from multivariate Cox proportional hazards regression, with clinical variates of onset age, gender, and white blood cell (WBC) count. All tests were two-tailed, and p-values < 0.05 were considered to be significant.

### Ethics declaration

This study was conducted in accordance with the Declaration of Helsinki guidelines. Written informed consent was obtained from all study participants or their guardians.

## Results

### Clinical characteristics of the patients

Among the 64 children with T-ALL, 45 were male and 19 were female, with a male to female ratio of 2.4:1. The median age of the patients at diagnosis was 12.1 years (range 1.4–17.4 years). The median WBC count was 69.8 × 10^9^ /L (range 0.6–1096 × 10^9^/L). Twenty-two (34.4%) patients had central nervous system (CNS) leukemia at diagnosis. The clinical features of this cohort are given in Table 1.

**Table 1:** Clinical characteristics of this T-ALL cohort.

	T-ALL	
	(n = 64)	
	n	%
**Gender**		
Male	45	70.3
Female	19	29.7
**Age (years)**		
1–9.9	24	37.5
≥ 10	40	62.5
**WBC (× 10**^**9**^**/L)**		
< 50	27	42.2
≥ 50	36	56.3
No data	1	1.6
**Protocol**		
TPOG 2002	49	76.6
TPOG 2013	15	23.4
**Early treatment response#**		
MRD positive	4	6.3
MRD negative	10	15.6
No data	50	78.1
**Subtype**		
TAL1	12	19.4
TAL2	4	6.3
TLX3	1	1.6
LMO1/LMO2	1	1.6
LMO2/LYL1	2	3.1
NKX2-1	2	3.1
HOXA	6	9.4
Other	17	26.6
No data	19	30.7
**Early T-cell precursor**		
Positive	7	10.9
Negative	29	45.3
No data	28	43.8
**TRG status**		
ABD	12	18.8
Deletion	24	37.5
No data	28	43.8
**CNS Leukemia**		
Positive	22	34.4
Negative	39	60.9
No data	3	4.7
**Bone marrow transplantation**		
Total patients	12	18.8
Living	5	7.8
Deceased	7	10.9
**Outcome**		
Death	24	37.5
Relapse	28	43.8
No event	36	56.3

### Landscape of genetic alterations in T-cell acute lymphoblastic leukemia

**Figure 1:**
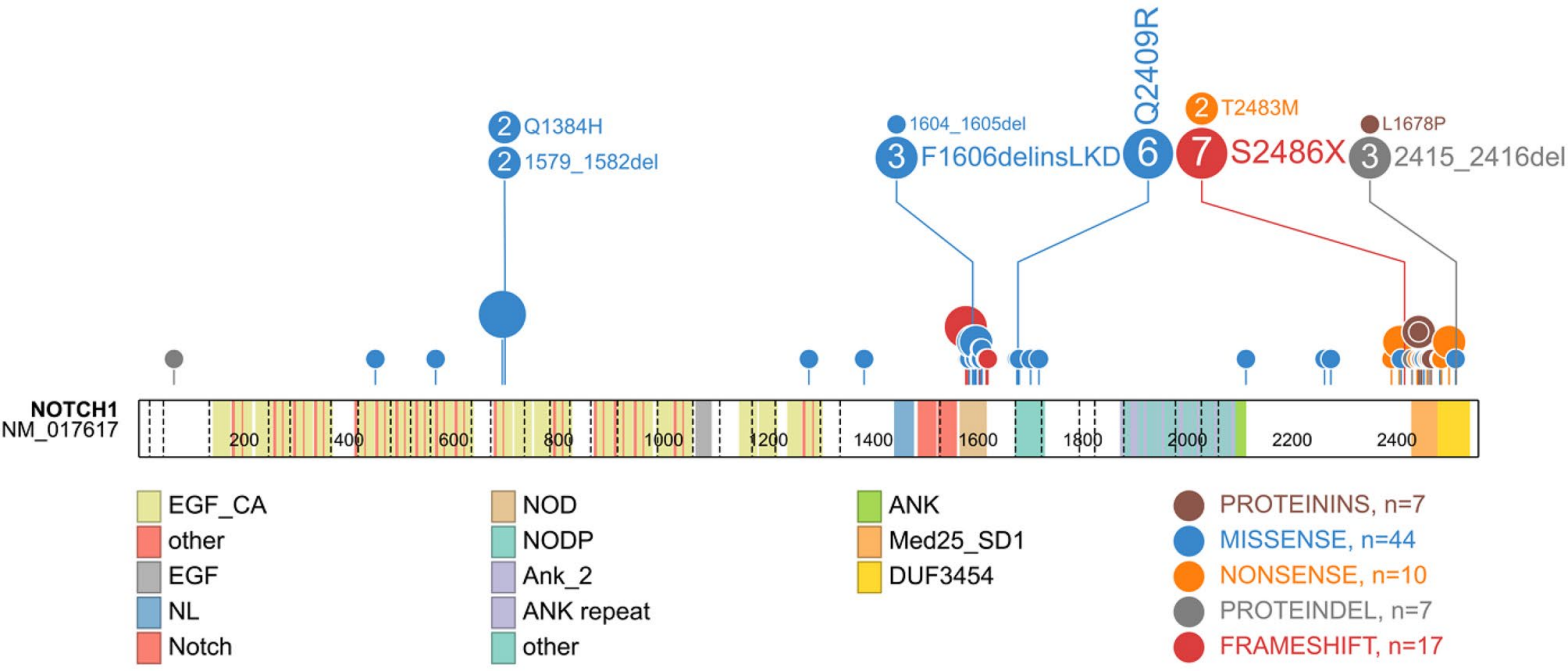
Genetic profiling of *NOTCH1* genes. Fifty-seven genetic mutations were detected in *NOTCH1* and 70.31% of patients carried *NOTCH1* mutations. *EGF_CA* calcium-binding EGF-like domain; Other: Ca^2+^ binding site; *EGF* EGF-like domain, *NL* domain found in Notch and Lin-12. *Notch* LNR domain. *NOD and NODP* NOTCH protein, *Ank_2* ankyrin repeats, *ANK repeat* ANK repeat, *ANK* ankyrin repeats, *Med25_SD1* mediator complex subunit 25 synapsin 1, *DUF3454* domain of unknown function (DUF3454).

**Figure 2:**
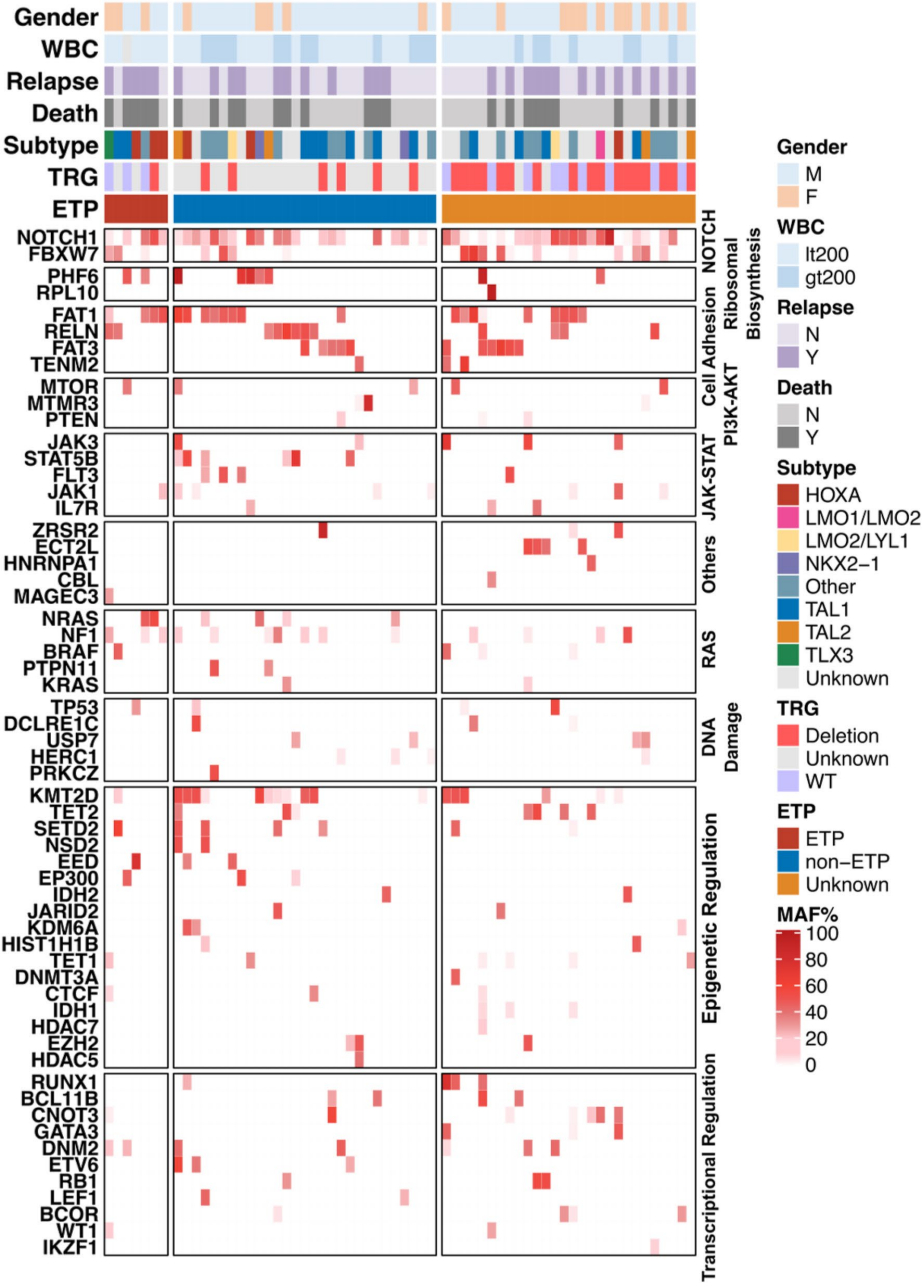
Genetic alteration profiling in 64 cases of T-cell acute lymphoblastic leukemia. Each column represents the genetic alterations of a single patient. *AF* alternative allele fraction, *TRG* T cell receptor-γ locus.

In all, 302 variants were identified in 60 genes, including 233 single nucleotide variants (SNVs) and 69 insertions and deletions (indels). These variants were all predicted to result in amino change and majority variants were single nucleotide variants (SNVs) (6.29% frameshift deletion, 8.61% frameshift insertion, 4.97% non-frameshift deletion, 2.32% non-frameshift insertion, 6.95% stop gain, and 70.86% nonsynonymous SNVs). The 64 samples showed a median number of 6 genetic lesions (range 1–17). Among 60 impacted genes, 13 had mutation frequencies > 10%, and 5 had > 20% carrier frequencies including *NOTCH1* (70.31%), *FAT1* (32.81%), *FBXW7* (28.13%), *KMT2D* (28.13%) and *NF1* (21.88%). As expected, the *NOTCH1* gene had the highest mutation rate, with the majority of the mutations detected in exon 26 (35.09%) and exon 34 (42.11%). The details of the *NOTCH1* mutations are shown in Fig. 1. Interestingly, protocadherins *FAT1* (32.81%) and *FAT3* (17.19%), and the ubiquitin ligase component *FBXW7* (28.13%) had high mutation frequencies in our cohort. Mutation frequencies of *PHF6* (14.06%), *DNM2* (10.94%), *DNMT3A* (4.69%), *CNOT3* (10.94%), and *WT1* (3.13%) were in the range of those previously reported. Mutations in epigenetic-related genes *KMT2D* (28.13%), *DNMT3A* (4.69%), and *EZH2* (4.69%) were detected in our patients. For T-ALL-related pathogenesis pathways, impacted genes involved in the *JAK-STAT* signaling pathways, such as *EP300* (4.69%), *STAT5B* (9.38%), *JAK1* (12.50%), *JAK3* (7.81%), *IL7R* (4.69%), and *PTPN11* (3.13%) had mutation frequencies in the range of 3%–13% and were observed in 23 cases (35.94%). Impacted genes involved in the *ErbB* signaling pathway were detected in 20 patients (31.25%), including *NRAS* (9.38%), *KRAS* (3.13%), *BRAF* (6.25%), *STAT5B* (9.38%), *CBL* (1.56%), and *MTOR* (7.81%). The genetic alterations in each of the gene expression subtypes are listed in Fig. 2. We observed a higher frequency of mutations in RAS signaling in ETP ALL cases (p = 0.04) (Fig. 2). The details of the sequencing results, including the gene list and mutational frequency, are listed in Supplementary Table 1.

### Paired samples show relapse-acquired mutations

**Figure 3:**
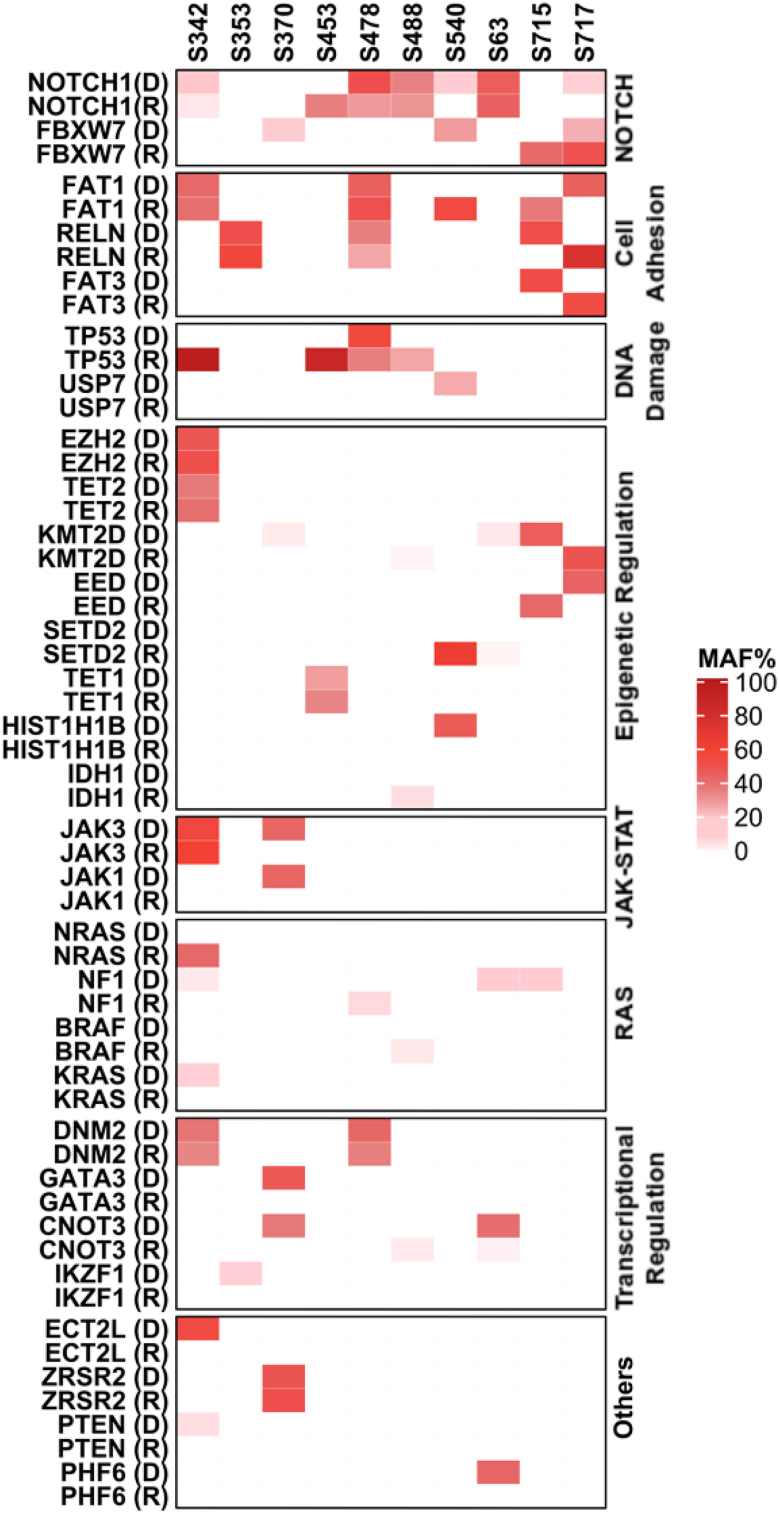
Mutation allele fractions in 29 genes differ between diagnosis and relapse in 10 patients. *MAF* mutation allele fraction.

When we compared the mutation status of 10 diagnosis-relapse pairs, the results showed that 29 genes exhibited mutation allele fraction (MAF) changes between diagnosis and relapse. The mutational status of diagnosis and relapse were quite different (Fig. 3). Noticeably, among these 29 genes, 70% of the patients maintained or acquired *NOTCH1* or *FBXW7* mutations during relapse. Both of these genes are involved in the *NOTCH* signaling pathway. In addition, 60% of the patients had obvious mutations in cell adhesion related genes after relapse. Finally, it is worth noting that 40% of the patients acquired *TP53* mutations in this process.

### The prognostic relevance of genetic alterations in T-ALL, when treated according to the TPOG-ALL protocols

Because *NOTCH1* and *FBXW7* are commonly discussed prognostic markers in childhood T-ALL, we tested these two genotypes against the prognosis. Although patients with both gene mutations showed slightly better 5-year event-free survival (EFS) and overall survival (OS), this was not significant.

**Figure 4:**
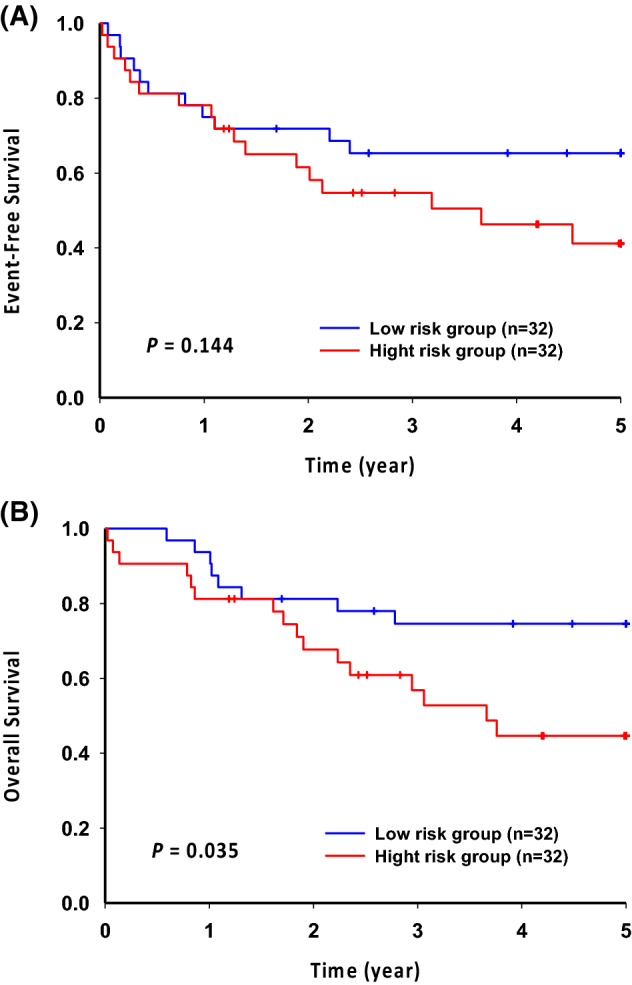
Event-free survival (**A**) and overall survival (**B**) of patients with T-cell acute lymphoblastic leukemia, stratified by genetic lesions in *NOTCH1*, *FBWX7*, *RAS,* and *PTEN.* Patients with *NOTCH1*/*FBXW7* mutations and *RAS*/*PTEN* germline were considered to be a low risk group (n = 32) and the others were classified as a high-risk group (n = 32).

The genetic profiles of *NOTCH1*, *FBWX7*, *RAS* and *PTEN* have been reported to be prognostic genotypes in T-ALL in both adults and children. Patients with *NOTCH1*/*FBXW7* mutations and *RAS/PTEN* germline have been considered as oncogenic low risk, whereas those with *NOTCH1*/*FBXW7* germline and *RAS*/*PTEN* germline or *NOTCH1*, *FBXW7, RAS*, and *PTEN* mutations are classified as high risk^37,38^. In this cohort, patients with *NOTCH1*/*FBXW7* mutations and *RAS*/*PTEN* germline had better 5-year EFS (65.3%; 95% CI 50.7%–84.2%) and OS (74.6%; 95% CI 60.8%–91.5%) than patients with other genetic combinations (EFS: 41.2%; 95% CI 26.0%–65.2%; OS: 44.7%; 95% CI 29.4%–67.8%), although only the 5-year OS has statistical significance (Fig. 4). In multivariate analysis of initial white cell counts, age of onset, and gender, patients with *NOTCH1*/*FBXW7* mutations and *RAS*/*PTEN* germline had significantly better 5-year OS than patients with other genetic combinations (adjusted HR 0.36; 95% CI 0.15–0.89, p = 0.0268).

## Discussion

Targeted sequencing is able to profile multiple genetic mutations, with different allele frequencies, at the same time. In this study, *NOTCH1* mutations were the most common genetic alterations, accounting for more than 70% of mutations, followed by the *JAK-STAT* pathway (35.9%), which may be suitable for targeted therapy. Patients with *NOTCH1*/*FBXW7* mutations and *RAS*/*PTEN* germline had a better 5-year EFS and OS than patients with other genetic combinations, although only the 5-year OS had statistical significance.

Some genetic alterations have been reported to be prognostic markers in childhood T-ALL^24,25,43–45^. The most common prognostic genetic alterations are to *NOTCH1* signaling pathways. Patients with *NOTCH1* mutations have previously been viewed as markers of good prognosis. However, prognostic impacts are not consistent in all related studies^23,24,26–29^. Two studies showed that the genetic alterations of *NOTCH1, FBXW7, PTEN*, and *RAS* were identified with prognostic value in adult T-ALL^37,46^. These oncogenic mutations, combined with MRD, might improve outcome prediction in cases of pediatric T-ALL when treated according to the protocol known as FRALLE2000T^38^. However, it was impossible to validate these oncogenic alterations in another 145 pediatric T-ALL patients treated in the UKALL 2003 trial^25^. There may be several reasons for the discrepancy between the two T-ALL cohorts. The UKALL cohort was relatively small, different molecular techniques were used and the results may have been interpreted differently. The incidence of *PTEN* abnormality was higher in the UKALL cohort (22%) than the FRALLE2000T cohort (14%) was. In our study, unfortunately, we lacked the MRD data for most patients, which would be required to validate its prognostic value. Another study from Taiwan, using Sanger sequencing, showed that *PHF6* was an independent prognostic marker, after multivariate analysis^47^. However, the authors did not investigate the prognostic value of mutations to *NOTCH1*/*FBXW7* and the *RAS*/*PTEN* germline in their cohort.

Another important strength of targeted sequencing is the ability to identify these targetable genetic alterations^22^. There are several possible targeted therapies for T-ALL, which have been investigated recently. For example, *NOTCH1* is the most common genetic alteration and there are available drugs that interfere with the activation of NOTCH1 by the r-secretase complex in childhood T-ALL^22,48–50^. Antibodies targeting this pathway also showed some promising preclinical results^51,52^. Most importantly, the *JAK-STAT* pathway might be the most promising target due to the availability of drugs focused on this pathway and the fact that one third of patients have alterations in this pathway^10,22,30,32,47^. Our findings suggest that the genotyping of *NOTCH1, FBXW7, RAS,* and *PTEN* should be considered for risk-directed therapies, alongside the response to induction chemotherapy, in future TPOG ALL protocols. In addition, further investigation of the mutations in the *JAK-STAT* pathway could facilitate possible target-based therapies. These approaches might increase the survival of childhood T-ALL in Taiwan. Currently, the sequencing of these genes might require NGS with targeted sequencing or whole exome sequencing (WES) if it is available.

The genetic frequency differed slightly from previous reports^22,30^; for example, *NOTCH1* was around 50% in most studies that used the methodology of Sanger sequencing^8,23,26,28,29,47^. When Liu et al. used WES and/or WGS to profile the genetic landscape of T-ALL, *NOTCH1* was found to be the most frequently mutated gene, with 264 sequence mutations, identified in 196 cases, and most mutations were in the heterodimerization domain (62.9%; 166/264) and the PEST domain (31.4%; 83/264). Of the 264 mutations, 116 (43.9%) were subclonal (AF < 30%)^20^. Our target sequencing data are similar to this, with a higher somatic mutational rate of *NOTCH1* due to subclonal mutations. Zhang et al. observed a high frequency of mutations resulting in aberrant cytokine receptor and RAS signaling, and alterations of genes with roles in hematopoietic and lymphoid development in ETP-ALL^19^. However, due to the smaller sample size of this cohort and the lack of adequate immunophenotypes to determine ETP-ALL in some of the patients, we were only able to conclude that alterations of the RAS pathway were higher in ETP-ALL patients in this study. In addition, we had ten patients with paired diagnostic and relapsed samples. One of the notable findings in our study was that four patients had acquired *TP53* mutations, which is compatible with our previous study^53^.

There are some limitations of this study. The first is that the cohort is relatively small, and complete genetic profiling might need a larger cohort. Second, some novel genetic pathways related to T-ALL were not designed in this target-seq panel, such as the MYC and FA pathways^20,54^. Therefore, we did not have a comprehensive genetic picture of our Taiwanese cohort; in the future, we intend to use WES. Targeted sequencing might also miss some other genetic alterations in T-ALL cases. Finally, most of the patients in this cohort lacked MRD data, following induction chemotherapy; therefore, we are not able to correlate the genetic alterations with MRD. To assess the prognostic value of mutations to *NOTCH1*/*FBXW7* and the *RAS*/*PTEN* germline, a larger prospective cohort is needed, to validate their association with MRD in TPOG protocols.

In conclusion, this study showed that amplicon-based next generation sequencing is able to identify the common genetic alterations in childhood T-ALL. Some of the identified genetic lesions may be suitable therapeutic targets and some might have prognostic value. The identification of *JAK-STAT* pathway alterations may also be useful additions for targeted therapy after induction in future TPOG ALL protocols^39^. A larger, prospective clinical trial is needed, coordinated with MRD data, to validate the clinical significance of key mutations in Taiwanese patients treated with TPOG protocols.

## Supplementary Information

Supplementary Information.

## Data Availability

The datasets generated during and/or analyzed during the current study are available from the corresponding author on reasonable request.
