# Supplementary material for: Targeted sequencing to identify genetic alterations and prognostic markers in pediatric T-cell acute lymphoblastic leukemia
Source: Sci Rep. 2021 Jan 12;11:769. doi: 10.1038/s41598-020-80613-6 (PMC7804301; doi:10.1038/s41598-020-80613-6)
Supplement: Supplementary file 1 — Supplementary Information. [file 41598_2020_80613_MOESM1_ESM.pdf]

Targeted sequencing to identify genetic alterations and prognostic markers in pediatric T-cell acute lymphoblastic leukemia

Ya-Hsuan Chang[1*], Chih-Hsiang Yu[2*], Shiann-Tarng Jou[3,4], Chien-Yu Lin[2], Kai-Hsin Lin[3,4], Meng-Yao Lu[3,4], Kang-Hsi Wu[5], Hsiu-Hao Chang[3,4], Dong-Tsamn Lin[3,4,6,7], Shu-Wha Lin[2,6], Hsuan-Yu Chen[1], Yung-Li Yang[3,6,7]

[1]Institute of Statistical Science Academia Sinica, Taipei, Taiwan;

[2]Departments of Clinical Laboratory Sciences and Medical Biotechnology, National Taiwan University, College of Medicine, Taipei, Taiwan;

[3]Department of Pediatrics, National Taiwan University Hospital, Taipei, Taiwan;

[4]Department of Pediatrics, College of Medicine, National Taiwan University, Taipei, Taiwan;

[5]Department of Pediatrics, Chung Shan Medical University Hospital and School of Medicine, Chung Shan Medical University, Taichung, Taiwan.;

[6]Department of Laboratory Medicine, National Taiwan University Hospital, Taipei, Taiwan;

[7]Department of Laboratory Medicine, College of Medicine, National Taiwan University, Taipei, Taiwan;

*These authors contributed equally to this article.

Corresponding authors: Yung-Li Yang, Department of Laboratory Medicine, National Taiwan University Hospital, Taipei, Taiwan, 100, No 7. Chung-Shan South Road, (yangyl92@ntu.edu.tw), Telephone: +886-2-23123456-71712, Fax: +886-2-23224263 or Hsuan-Yu Chen, Institute of Statistical Science Academia Sinica, Taipei, Taiwan, 128 Academia Road, Section 2, Nankang, Taipei 11529, Taiwan, (hychen@stat.sinica.edu.tw) Telephone: +886-2-27875600, Fax: +886-2-27886833

Supplementary Table 1: Mutational spectrum in 64 T-cell acute lymphoblastic leukemia.

Chr	Position	Reference Allele	Alternative allele	Gene	Mutation type	RefSeq	AA change	Carrier frequency	Clinvar_annotation
chr1	2075748	G	A	*PRKCZ*	nonsynonymous SNV	NM_001242874	p.G70S	1.56%	NA
chr1	11190698	GTGGTGGCGGC	-	*MTOR*	nonframeshift deletion	NM_004958	p.1830_1834del	3.13%	Benign
chr1	11217231	A	G	*MTOR*	nonsynonymous SNV	NM_004958	p.C1483R	1.56%	Pathogenic/Likely_pathoge
chr1	11272529	G	A	*MTOR*	nonsynonymous SNV	NM_004958	p.A1134V	3.13%	NA
chr1	11273494	T	C	*MTOR*	nonsynonymous SNV	NM_004958	p.M1083V	1.56%	NA
chr1	65305426	G	C	*JAK1*	nonsynonymous SNV	NM_001321852	p.T901R	1.56%	NA
chr1	65306941	C	T	*JAK1*	nonsynonymous SNV	NM_001321852	p.R879H	1.56%	NA
chr1	65306942	G	T	*JAK1*	nonsynonymous SNV	NM_001321852	p.R879S	1.56%	NA
chr1	65310517	C	T	*JAK1*	nonsynonymous SNV	NM_001321852	p.R724H	1.56%	NA
chr1	65339111	T	-	*JAK1*	frameshift deletion	NM_001321852	p.K142fs	7.81%	NA
chr1	115258747	C	G	*NRAS*	nonsynonymous SNV	NM_002524	p.G12A	1.56%	Pathogenic
chr1	115258747	C	T	*NRAS*	nonsynonymous SNV	NM_002524	p.G12D	1.56%	Pathogenic
chr1	115258748	C	T	*NRAS*	nonsynonymous SNV	NM_002524	p.G12S	4.69%	Pathogenic
chr1	115258748	C	A	*NRAS*	nonsynonymous SNV	NM_002524	p.G12C	1.56%	Pathogenic
chr2	25467089	G	A	*DNMT3A*	nonsynonymous SNV	NM_001320893	p.R444W	1.56%	NA
chr2	209113096	A	T	*IDH1*	nonsynonymous SNV	NM_001282386	p.D137E	4.69%	NA
chr3	47058647	A	T	*SETD2*	nonsynonymous SNV	NM_001349370	p.I2500N	1.56%	NA
chr3	47058736	G	T	*SETD2*	nonsynonymous SNV	NM_001349370	p.H2470Q	1.56%	NA
chr3	47125437	C	T	*SETD2*	nonsynonymous SNV	NM_001349370	p.E1901K	1.56%	NA
chr3	47163212	C	G	*SETD2*	nonsynonymous SNV	NM_001349370	p.E928Q	1.56%	NA
chr3	47163689	C	T	*SETD2*	nonsynonymous SNV	NM_001349370	p.E769K	3.13%	NA
chr3	47163843	C	T	*SETD2*	nonsynonymous SNV	NM_001349370	p.M717I	1.56%	not_provided
chr3	47165569	G	A	*SETD2*	nonsynonymous SNV	NM_001349370	p.P142L	1.56%	Benign
chr4	1906007	A	G	*NSD2*	nonsynonymous SNV	NM_133334	p.N221S	1.56%	NA
chr4	1936899	C	A	*NSD2*	nonsynonymous SNV	NM_133334	p.H528Q	1.56%	Likely_benign
chr4	106155392	T	A	*TET2*	nonsynonymous SNV	NM_001127208	p.V98D	1.56%	NA
chr4	106157703	T	G	*TET2*	nonsynonymous SNV	NM_001127208	p.F868L	3.13%	not_provided
chr4	106158550	G	T	*TET2*	stopgain	NM_017628	p.E1151X	4.69%	not_provided
chr4	106158578	G	A	*TET2*	nonsynonymous SNV	NM_017628	p.G1160E	1.56%	not_provided
chr4	108985522	T	A	*LEF1*	stopgain	NM_001130713	p.K351X	1.56%	NA
chr4	108991911	-	AC	*LEF1*	frameshift insertion	NM_001130713	p.R314fs	1.56%	NA
chr4	108999438	-	GGGGAGA	*LEF1*	frameshift insertion	NM_001130713	p.N288fs	1.56%	NA
chr4	153244092	G	A	*FBXW7*	nonsynonymous SNV	NM_001013415	p.R571W	1.56%	NA
chr4	153244148	C	-	*FBXW7*	frameshift deletion	NM_001013415	p.G552fs	3.13%	NA
chr4	153247366	C	T	*FBXW7*	nonsynonymous SNV	NM_001013415	p.R361Q	3.13%	Likely_pathogenic
chr4	153249366	T	C	*FBXW7*	nonsynonymous SNV	NM_001013415	p.E353G	1.56%	NA
chr4	153249384	C	T	*FBXW7*	nonsynonymous SNV	NM_001013415	p.R347H	6.25%	Likely_pathogenic
chr4	153249385	G	A	*FBXW7*	nonsynonymous SNV	NM_001013415	p.R347C	4.69%	Likely_pathogenic
chr4	153249468	C	A	*FBXW7*	nonsynonymous SNV	NM_001013415	p.G319V	1.56%	NA
chr4	153249504	C	G	*FBXW7*	nonsynonymous SNV	NM_001013415	p.W307S	1.56%	NA
chr4	153249510	C	A	*FBXW7*	nonsynonymous SNV	NM_001013415	p.G305V	1.56%	NA
chr4	153303426	G	C	*FBXW7*	nonsynonymous SNV	NM_001013415	p.A21G	1.56%	NA
chr4	153303427	C	T	*FBXW7*	nonsynonymous SNV	NM_001013415	p.A21T	1.56%	NA
chr4	153332579	T	A	*FBXW7*	nonsynonymous SNV	NM_033632	p.D126V	3.13%	NA
chr4	153332605	CTC	-	*FBXW7*	nonframeshift deletion	NM_033632	p.117_117del	1.56%	NA
chr4	153332619	C	A	*FBXW7*	stopgain	NM_033632	p.E113X	1.56%	NA
chr4	187509906	G	C	*FAT1*	nonsynonymous SNV	NM_005245	p.P4536R	1.56%	NA
chr4	187510265	A	T	*FAT1*	nonsynonymous SNV	NM_005245	p.D4416E	1.56%	NA
chr4	187519226	C	T	*FAT1*	nonsynonymous SNV	NM_005245	p.V4053I	1.56%	NA
chr4	187521337	T	C	*FAT1*	nonsynonymous SNV	NM_005245	p.T3940A	3.13%	NA
chr4	187524503	A	T	*FAT1*	nonsynonymous SNV	NM_005245	p.I3726N	1.56%	NA
chr4	187524600	C	T	*FAT1*	nonsynonymous SNV	NM_005245	p.V3694I	1.56%	NA
chr4	187525704	C	G	*FAT1*	nonsynonymous SNV	NM_005245	p.V3459L	1.56%	NA
chr4	187535411	T	C	*FAT1*	nonsynonymous SNV	NM_005245	p.N3055D	1.56%	NA
chr4	187538271	T	A	*FAT1*	nonsynonymous SNV	NM_005245	p.K2988I	6.25%	NA
chr4	187538912	G	A	*FAT1*	nonsynonymous SNV	NM_005245	p.T2943M	1.56%	NA
chr4	187539275	A	G	*FAT1*	nonsynonymous SNV	NM_005245	p.L2822P	1.56%	NA
chr4	187539645	G	C	*FAT1*	nonsynonymous SNV	NM_005245	p.Q2699E	1.56%	NA
chr4	187539941	T	C	*FAT1*	nonsynonymous SNV	NM_005245	p.K2600R	1.56%	NA
chr4	187540027	C	G	*FAT1*	nonsynonymous SNV	NM_005245	p.K2571N	1.56%	NA
chr4	187541475	C	T	*FAT1*	nonsynonymous SNV	NM_005245	p.V2089I	1.56%	NA
chr4	187541711	T	A	*FAT1*	nonsynonymous SNV	NM_005245	p.E2010V	3.13%	NA
chr4	187549718	T	A	*FAT1*	nonsynonymous SNV	NM_005245	p.D1508V	1.56%	NA
chr4	187584696	C	T	*FAT1*	nonsynonymous SNV	NM_005245	p.D1113N	3.13%	NA
chr4	187630615	G	A	*FAT1*	nonsynonymous SNV	NM_005245	p.L123F	1.56%	NA
chr5	35871238	C	T	*IL7R*	nonsynonymous SNV	NM_002185	p.H154Y	1.56%	not_provided
chr5	35874566	-	GGG	*IL7R*	nonframeshift insertion	NM_002185	p.I241delinsMG	1.56%	NA
chr5	35874584	-	GCGCCCTTG	*IL7R*	nonframeshift insertion	NM_002185	p.I247delinsMRPC	1.56%	NA
chr5	167303149	A	G	*TENM2*	nonsynonymous SNV	NM_001122679	p.N221D	1.56%	NA
chr5	167379643	C	G	*TENM2*	nonsynonymous SNV	NM_001080428	p.P64A	1.56%	NA
chr5	167674863	C	T	*TENM2*	nonsynonymous SNV	NM_001080428	p.R2068W	1.56%	NA
chr6	15496408	G	C	*JARID2*	nonsynonymous SNV	NM_001267040	p.G146R	1.56%	NA
chr6	15501529	G	C	*JARID2*	nonsynonymous SNV	NM_001267040	p.Q607H	1.56%	NA
chr6	27834630	CTT	-	*HIST1H1B*	nonframeshift deletion	NM_005322	p.226_226del	1.56%	NA
chr6	27835235	T	C	*HIST1H1B*	nonsynonymous SNV	NM_005322	p.T25A	1.56%	NA
chr6	139202299	G	A	*ECT2L*	nonsynonymous SNV	NM_001195037	p.S624N	1.56%	NA
chr6	139206697	A	G	*ECT2L*	nonsynonymous SNV	NM_001195037	p.I695V	1.56%	NA
chr6	139208021	C	G	*ECT2L*	nonsynonymous SNV	NM_001195037	p.H763D	1.56%	NA
chr6	139223733	G	A	*ECT2L*	nonsynonymous SNV	NM_001195037	p.R895Q	1.56%	NA
chr7	50450372	G	T	*IKZF1*	nonsynonymous SNV	NM_001220767	p.D99Y	1.56%	NA
chr7	103124173	T	C	*RELN*	nonsynonymous SNV	NM_005045	p.T3370A	3.13%	Likely_benign

chr7	103138354	G	A	*RELN*	nonsynonymous SNV	NM_005045	p.R2955C	3.13%	Conflicting_interpretations_of_pathogenicity
chr7	103179743	G	A	*RELN*	nonsynonymous SNV	NM_005045	p.T2321M	6.25%	Likely_benign
chr7	103207075	C	T	*RELN*	nonsynonymous SNV	NM_005045	p.A1574T	1.56%	Conflicting_interpretations_of_pathogenicity
chr7	103234329	T	G	*RELN*	nonsynonymous SNV	NM_005045	p.N1238H	3.13%	Benign/Likely_benign
chr7	103368614	C	T	*RELN*	nonsynonymous SNV	NM_005045	p.A233T	1.56%	Uncertain_significance
chr7	140434513	A	T	*BRAF*	nonsynonymous SNV	NM_004333	p.S729T	3.13%	NA
chr7	140481451	G	C	*BRAF*	nonsynonymous SNV	NM_004333	p.P453A	1.56%	NA
chr7	140500178	C	A	*BRAF*	nonsynonymous SNV	NM_004333	p.A322S	1.56%	NA
chr7	148506443	C	T	*EZH2*	nonsynonymous SNV	NM_001203249	p.R634H	1.56%	Likely_pathogenic
chr7	148506467	G	A	*EZH2*	nonsynonymous SNV	NM_001203249	p.A626V	1.56%	NA
chr7	148523708	C	T	*EZH2*	nonsynonymous SNV	NM_152998	p.E210K	1.56%	Uncertain_significance
chr7	148543614	-	T	*EZH2*	frameshift insertion	NM_001203247	p.I65fs	1.56%	NA
chr7	148543619	-	C	*EZH2*	frameshift insertion	NM_001203247	p.R63fs	1.56%	NA
chr9	139390649	-	G	*NOTCH1*	frameshift insertion	NM_017617	p.P2514fs	1.56%	NA
chr9	139390649	AG	-	*NOTCH1*	frameshift deletion	NM_017617	p.P2514fs	4.69%	Uncertain_significance
chr9	139390653	G	A	*NOTCH1*	nonsynonymous SNV	NM_017617	p.S2513F	1.56%	NA
chr9	139390690	G	A	*NOTCH1*	stopgain	NM_017617	p.Q2501X	3.13%	NA
chr9	139390734	G	T	*NOTCH1*	stopgain	NM_017617	p.S2486X	1.56%	NA
chr9	139390743	G	A	*NOTCH1*	nonsynonymous SNV	NM_017617	p.T2483M	1.56%	NA
chr9	139390793	-	TTGGGACT	*NOTCH1*	frameshift insertion	NM_017617	p.T2466fs	1.56%	NA
chr9	139390805	G	-	*NOTCH1*	frameshift deletion	NM_017617	p.P2462fs	1.56%	NA
chr9	139390816	G	A	*NOTCH1*	stopgain	NM_017617	p.Q2459X	1.56%	NA
chr9	139390834	C	T	*NOTCH1*	nonsynonymous SNV	NM_017617	p.V2453M	1.56%	NA
chr9	139390847	-	C	*NOTCH1*	frameshift insertion	NM_017617	p.P2448fs	1.56%	NA
chr9	139390855	GTGG	-	*NOTCH1*	frameshift deletion	NM_017617	p.P2445fs	1.56%	NA
chr9	139390863	-	GG	*NOTCH1*	frameshift insertion	NM_017617	p.V2443fs	3.13%	NA
chr9	139390864	-	GA	*NOTCH1*	frameshift insertion	NM_017617	p.V2443fs	1.56%	NA
chr9	139390873	G	A	*NOTCH1*	stopgain	NM_017617	p.Q2440X	1.56%	NA
chr9	139390902	C	-	*NOTCH1*	frameshift deletion	NM_017617	p.G2430fs	1.56%	NA
chr9	139390945	G	A	*NOTCH1*	stopgain	NM_017617	p.Q2416X	3.13%	NA
chr9	139390945	GTG	-	*NOTCH1*	nonframeshift deletion	NM_017617	p.2415_2416del	10.94%	Likely_benign
chr9	139390965	T	C	*NOTCH1*	nonsynonymous SNV	NM_017617	p.Q2409R	1.56%	NA
chr9	139390975	G	A	*NOTCH1*	stopgain	NM_017617	p.Q2406X	3.13%	NA
chr9	139391020	G	A	*NOTCH1*	stopgain	NM_017617	p.Q2391X	1.56%	NA
chr9	139391367	T	A	*NOTCH1*	nonsynonymous SNV	NM_017617	p.H2275L	1.56%	NA
chr9	139391403	C	T	*NOTCH1*	nonsynonymous SNV	NM_017617	p.R2263Q	1.56%	Uncertain_significance
chr9	139391853	A	C	*NOTCH1*	nonsynonymous SNV	NM_017617	p.L2113R	1.56%	NA
chr9	139397648	A	G	*NOTCH1*	nonsynonymous SNV	NM_017617	p.I1718T	1.56%	NA
chr9	139397696	A	G	*NOTCH1*	nonsynonymous SNV	NM_017617	p.F1702S	1.56%	NA
chr9	139397762	A	T	*NOTCH1*	nonsynonymous SNV	NM_017617	p.I1680N	1.56%	NA
chr9	139397768	A	G	*NOTCH1*	nonsynonymous SNV	NM_017617	p.L1678P	9.38%	NA
chr9	139397774	A	T	*NOTCH1*	nonsynonymous SNV	NM_017617	p.V1676D	1.56%	NA
chr9	139399283	GTAGTA	-	*NOTCH1*	nonframeshift deletion	NM_017617	p.1619_1620del	1.56%	NA
chr9	139399286	-	TGTGGCGA	*NOTCH1*	frameshift insertion	NM_017617	p.Y1619fs	1.56%	NA
chr9	139399292	-	TTT	*NOTCH1*	nonframeshift insertion	NM_017617	p.F1617delinsLN	1.56%	NA
chr9	139399317	T	G	*NOTCH1*	nonsynonymous SNV	NM_017617	p.D1609A	1.56%	NA
chr9	139399318	C	G	*NOTCH1*	nonsynonymous SNV	NM_017617	p.D1609H	1.56%	NA
chr9	139399321	G	C	*NOTCH1*	nonsynonymous SNV	NM_017617	p.R1608G	1.56%	NA
chr9	139399323	TTG	-	*NOTCH1*	nonframeshift deletion	NM_017617	p.1606_1607del	1.56%	NA
chr9	139399325	-	AGGCCA	*NOTCH1*	nonframeshift insertion	NM_017617	p.F1606delinsFGL	1.56%	NA
chr9	139399325	-	TCTTTT	*NOTCH1*	nonframeshift insertion	NM_017617	p.F1606delinsLKD	1.56%	NA
chr9	139399329	ACC	-	*NOTCH1*	nonframeshift deletion	NM_017617	p.1604_1605del	1.56%	NA
chr9	139399332	A	C	*NOTCH1*	nonsynonymous SNV	NM_017617	p.V1604G	1.56%	NA
chr9	139399350	C	G	*NOTCH1*	nonsynonymous SNV	NM_017617	p.R1598P	3.13%	NA
chr9	139399353	C	A	*NOTCH1*	nonsynonymous SNV	NM_017617	p.S1597I	1.56%	Uncertain_significance
chr9	139399356	A	T	*NOTCH1*	nonsynonymous SNV	NM_017617	p.L1596H	1.56%	NA
chr9	139399365	A	G	*NOTCH1*	nonsynonymous SNV	NM_017617	p.L1593P	3.13%	NA
chr9	139399368	A	G	*NOTCH1*	nonsynonymous SNV	NM_017617	p.F1592S	4.69%	NA
chr9	139399369	A	G	*NOTCH1*	nonsynonymous SNV	NM_017617	p.F1592L	1.56%	NA
chr9	139399389	A	T	*NOTCH1*	nonsynonymous SNV	NM_017617	p.L1585Q	1.56%	NA
chr9	139399399	GCGGCATCA	-	*NOTCH1*	nonframeshift deletion	NM_017617	p.1579_1582del	1.56%	NA
chr9	139399409	CAC	-	*NOTCH1*	nonframeshift deletion	NM_017617	p.1578_1578del	4.69%	NA
chr9	139400196	C	G	*NOTCH1*	nonsynonymous SNV	NM_017617	p.Q1384H	1.56%	NA
chr9	139401233	C	T	*NOTCH1*	nonsynonymous SNV	NM_017617	p.R1279H	1.56%	Benign
chr9	139409073	C	G	*NOTCH1*	nonsynonymous SNV	NM_017617	p.G699A	3.13%	NA
chr9	139409074	C	A	*NOTCH1*	nonsynonymous SNV	NM_017617	p.G699C	3.13%	NA
chr9	139409088	T	A	*NOTCH1*	nonsynonymous SNV	NM_017617	p.E694V	6.25%	NA
chr9	139410139	T	C	*NOTCH1*	nonsynonymous SNV	NM_017617	p.I567V	1.56%	Uncertain_significance
chr9	139412291	C	T	*NOTCH1*	nonsynonymous SNV	NM_017617	p.D452N	1.56%	NA
chr9	139418371	G	-	*NOTCH1*	frameshift deletion	NM_017617	p.P67fs	1.56%	NA
chr10	8106004	G	A	*GATA3*	nonsynonymous SNV	NM_001002295	p.R276Q	1.56%	NA
chr10	8106036	G	A	*GATA3*	nonsynonymous SNV	NM_001002295	p.A287T	1.56%	NA
chr10	8115822	T	A	*GATA3*	nonsynonymous SNV	NM_001002295	p.S391T	1.56%	NA
chr10	8115922	-	CCCGCCATCCAG	*GATA3*	frameshift insertion	NM_001002295	p.H424fs	1.56%	NA
chr10	14951099	CTT	-	*DCLRE1C*	nonframeshift deletion	NM_001289076	p.347_348del	1.56%	Conflicting_interpretations_of_pathogenicity
chr10	14951152	C	T	*DCLRE1C*	nonsynonymous SNV	NM_001289076	p.R330H	1.56%	NA
chr10	14996006	T	A	*DCLRE1C*	nonsynonymous SNV	NM_001033855	p.S2C	1.56%	NA
chr10	70332613	T	C	*TET1*	nonsynonymous SNV	NM_030625	p.I173T	1.56%	NA
chr10	70332865	C	-	*TET1*	frameshift deletion	NM_030625	p.T257fs	1.56%	NA
chr10	70333787	G	A	*TET1*	nonsynonymous SNV	NM_030625	p.M564I	1.56%	NA
chr10	70405994	G	C	*TET1*	nonsynonymous SNV	NM_030625	p.E1170Q	1.56%	NA

chr10	70451218	A	T	*TET1*	nonsynonymous SNV	NM_030625	p.I2020F	4.69%	NA
chr10	89653840	C	A	*PTEN*	stopgain	NM_000314	p.Y46X	1.56%	Pathogenic
chr10	89711902	TA	-	*PTEN*	frameshift deletion	NM_000314	p.Y174fs	1.56%	NA
chr10	89720836	T	A	*PTEN*	nonsynonymous SNV	NM_000314	p.N329K	1.56%	NA
chr11	32417908	-	CGACCGTA	*WT1*	frameshift insertion	NM_000378	p.A365fs	1.56%	NA
chr11	32439189	TATAAA	-	*WT1*	nonframeshift deletion	NM_000378	p.293_295del	1.56%	NA
chr11	85956317	C	T	*EED*	nonsynonymous SNV	NM_001308007	p.P16S	1.56%	NA
chr11	85979586	G	A	*EED*	nonsynonymous SNV	NM_001308007	p.D317N	1.56%	NA
chr11	85988147	G	C	*EED*	nonsynonymous SNV	NM_001330334	p.W284C	1.56%	NA
chr11	92086100	C	G	*FAT3*	nonsynonymous SNV	NM_001008781	p.F274L	1.56%	NA
chr11	92087265	G	C	*FAT3*	nonsynonymous SNV	NM_001008781	p.D663H	1.56%	NA
chr11	92087587	C	T	*FAT3*	nonsynonymous SNV	NM_001008781	p.T770M	1.56%	NA
chr11	92531314	G	A	*FAT3*	nonsynonymous SNV	NM_001008781	p.G1712E	1.56%	NA
chr11	92532352	G	A	*FAT3*	nonsynonymous SNV	NM_001008781	p.R2058H	3.13%	NA
chr11	92532481	C	G	*FAT3*	nonsynonymous SNV	NM_001008781	p.T2101S	1.56%	NA
chr11	92533462	G	A	*FAT3*	nonsynonymous SNV	NM_001008781	p.R2428Q	1.56%	NA
chr11	92534344	C	G	*FAT3*	nonsynonymous SNV	NM_001008781	p.A2722G	1.56%	NA
chr11	92534453	A	G	*FAT3*	nonsynonymous SNV	NM_001008781	p.I2758M	1.56%	NA
chr11	92534497	G	A	*FAT3*	nonsynonymous SNV	NM_001008781	p.R2773H	4.69%	NA
chr11	92570848	T	A	*FAT3*	nonsynonymous SNV	NM_001008781	p.L3415H	1.56%	NA
chr11	92577865	C	T	*FAT3*	nonsynonymous SNV	NM_001008781	p.R3778C	1.56%	NA
chr11	119170339	C	T	*CBL*	nonsynonymous SNV	NM_005188	p.L857F	1.56%	Likely_benign
chr12	11992223	-	GGGCCT	*ETV6*	stopgain	NM_001987	p.R105delinsRAX	1.56%	NA
chr12	12037451	A	G	*ETV6*	nonsynonymous SNV	NM_001987	p.E361G	1.56%	NA
chr12	12037492	G	A	*ETV6*	nonsynonymous SNV	NM_001987	p.G375R	1.56%	NA
chr12	12043929	-	CTGT	*ETV6*	frameshift insertion	NM_001987	p.H436fs	1.56%	NA
chr12	25398284	C	A	*KRAS*	nonsynonymous SNV	NM_004985	p.G12V	3.13%	Pathogenic
chr12	48185701	T	C	*HDAC7*	nonsynonymous SNV	NM_001098416	p.S552G	1.56%	NA
chr12	49420078	C	T	*KMT2D*	nonsynonymous SNV	NM_003482	p.R5224H	1.56%	Conflicting_interpretations _of_pathogenicity
chr12	49420995	-	G	*KMT2D*	frameshift insertion	NM_003482	p.P4918fs	1.56%	NA
chr12	49425230	G	A	*KMT2D*	nonsynonymous SNV	NM_003482	p.R4420W	1.56%	NA
chr12	49425818	C	T	*KMT2D*	nonsynonymous SNV	NM_003482	p.A4224T	1.56%	NA
chr12	49426772	GCT	-	*KMT2D*	nonframeshift deletion	NM_003482	p.3905_3906del	4.69%	NA
chr12	49427364	G	C	*KMT2D*	nonsynonymous SNV	NM_003482	p.S3708R	1.56%	Uncertain_significance
chr12	49431306	TGC	-	*KMT2D*	nonframeshift deletion	NM_003482	p.3277_3278del	1.56%	NA
chr12	49434005	G	-	*KMT2D*	frameshift deletion	NM_003482	p.P2516fs	1.56%	NA
chr12	49434014	C	-	*KMT2D*	frameshift deletion	NM_003482	p.G2513fs	1.56%	NA
chr12	49434630	T	C	*KMT2D*	nonsynonymous SNV	NM_003482	p.D2308G	1.56%	NA
chr12	49434689	C	A	*KMT2D*	nonsynonymous SNV	NM_003482	p.K2288N	1.56%	NA
chr12	49434910	A	T	*KMT2D*	nonsynonymous SNV	NM_003482	p.S2215T	1.56%	Likely_benign
chr12	49436954	C	T	*KMT2D*	nonsynonymous SNV	NM_003482	p.G1850D	1.56%	NA
chr12	49437445	T	C	*KMT2D*	nonsynonymous SNV	NM_003482	p.R1814G	1.56%	NA
chr12	49438285	A	C	*KMT2D*	nonsynonymous SNV	NM_003482	p.C1662G	1.56%	NA
chr12	49440464	T	C	*KMT2D*	nonsynonymous SNV	NM_003482	p.D1449G	1.56%	NA
chr12	49443539	C	G	*KMT2D*	nonsynonymous SNV	NM_003482	p.A1278P	1.56%	NA
chr12	49443827	A	G	*KMT2D*	nonsynonymous SNV	NM_003482	p.C1182R	1.56%	NA
chr12	49445732	C	G	*KMT2D*	nonsynonymous SNV	NM_003482	p.E578D	4.69%	NA
chr12	49446022	C	A	*KMT2D*	nonsynonymous SNV	NM_003482	p.A482S	1.56%	NA
chr12	49446441	-	A	*KMT2D*	frameshift insertion	NM_003482	p.L388fs	1.56%	NA
chr12	54676958	G	A	*HNRNPA1*	nonsynonymous SNV	NM_031157	p.G283R	1.56%	NA
chr12	112915482	A	G	*PTPN11*	nonsynonymous SNV	NM_001330437	p.D294G	1.56%	NA
chr12	112926875	T	C	*PTPN11*	nonsynonymous SNV	NM_001330437	p.S503P	1.56%	NA
chr13	28592629	T	C	*FLT3*	nonsynonymous SNV	NM_004119	p.D839G	1.56%	Likely_pathogenic
chr13	28592642	C	A	*FLT3*	nonsynonymous SNV	NM_004119	p.D835Y	1.56%	Pathogenic
chr13	28608500	T	C	*FLT3*	nonsynonymous SNV	NM_004119	p.I548V	1.56%	NA
chr13	28609652	G	A	*FLT3*	nonsynonymous SNV	NM_004119	p.T526M	1.56%	NA
chr13	28636182	C	T	*FLT3*	nonsynonymous SNV	NM_004119	p.G64R	1.56%	not_provided
chr13	48881451	C	T	*RB1*	nonsynonymous SNV	NM_000321	p.T58I	1.56%	Uncertain_significance
chr13	48941649	G	A	*RB1*	nonsynonymous SNV	NM_000321	p.R320Q	1.56%	Uncertain_significance
chr13	49039470	C	G	*RB1*	nonsynonymous SNV	NM_000321	p.L819V	1.56%	Benign/Likely_benign
chr14	99641759	G	A	*BCL11B*	nonsynonymous SNV	NM_001282238	p.R400C	1.56%	NA
chr14	99641824	G	A	*BCL11B*	nonsynonymous SNV	NM_001282238	p.T378M	1.56%	NA
chr14	99641855	T	C	*BCL11B*	nonsynonymous SNV	NM_001282238	p.S368G	1.56%	NA
chr14	99723871	-	G	*BCL11B*	frameshift insertion	NM_001282237	p.D121fs	1.56%	NA
chr15	63966930	C	T	*HERC1*	nonsynonymous SNV	NM_003922	p.R2486K	1.56%	NA
chr15	63986203	T	-	*HERC1*	frameshift deletion	NM_003922	p.K1878fs	4.69%	NA
chr15	90628130	T	C	*IDH2*	nonsynonymous SNV	NM_001290114	p.M267V	1.56%	NA
chr15	90631934	C	T	*IDH2*	nonsynonymous SNV	NM_001290114	p.R10Q	1.56%	Pathogenic
chr16	8989571	-	ACCCGC	*USP7*	nonframeshift insertion	NM_001286457	p.I933delinsMRV	1.56%	NA
chr16	9009161	CTATCAGAC	-	*USP7*	nonframeshift deletion	NM_001286457	p.324_327del	1.56%	NA
chr16	9009170	-	GA	*USP7*	frameshift insertion	NM_001286457	p.R324fs	1.56%	NA
chr16	9010980	-	TTCAGTCCGCTT TATTTACC	*USP7*	stopgain	NM_001286457	p.S236_S237delinsGKX	1.56%	NA
chr16	67645338	-	A	*CTCF*	frameshift insertion	NM_006565	p.A201fs	1.56%	Pathogenic
chr16	67645381	G	T	*CTCF*	stopgain	NM_006565	p.E216X	1.56%	NA
chr16	67646013	AC	-	*CTCF*	frameshift deletion	NM_006565	p.N314fs	1.56%	NA
chr17	7578181	G	-	*TP53*	frameshift deletion	NM_001126115	p.P91fs	1.56%	NA
chr17	7578237	C	A	*TP53*	nonsynonymous SNV	NM_001126115	p.E72D	1.56%	NA
chr17	7578413	C	T	*TP53*	nonsynonymous SNV	NM_001126115	p.V41M	1.56%	Pathogenic/Likely_pathoge
chr17	7579377	G	A	*TP53*	stopgain	NM_001126118	p.Q65X	1.56%	Pathogenic
chr17	7579705	C	T	*TP53*	nonsynonymous SNV	NM_000546	p.V31I	1.56%	Conflicting_interpretations _of_pathogenicity
chr17	29548962	-	T	*NF1*	frameshift insertion	NM_001128147	p.Y579fs	1.56%	Likely_pathogenic

chr17	29553507	A	-	*NF1*	frameshift deletion	NM_000267	p.K686fs	1.56%	NA
chr17	29553515	-	GT	*NF1*	frameshift insertion	NM_000267	p.E688fs	4.69%	NA
chr17	29553538	G	A	*NF1*	stopgain	NM_000267	p.W696X	1.56%	NA
chr17	29553550	C	G	*NF1*	nonsynonymous SNV	NM_000267	p.T700S	9.38%	NA
chr17	29554281	T	C	*NF1*	nonsynonymous SNV	NM_000267	p.I766T	6.25%	NA
chr17	29677260	CTT	-	*NF1*	nonframeshift deletion	NM_000267	p.2440_2440del	1.56%	Uncertain_significance
chr17	29679313	G	T	*NF1*	nonsynonymous SNV	NM_000267	p.G2478V	1.56%	NA
chr17	40353791	T	C	*STAT5B*	nonsynonymous SNV	NM_012448	p.M777V	1.56%	NA
chr17	40354410	C	A	*STAT5B*	nonsynonymous SNV	NM_012448	p.A729S	1.56%	Likely_benign
chr17	40359729	T	G	*STAT5B*	nonsynonymous SNV	NM_012448	p.N642H	6.25%	NA
chr17	42163994	G	A	*HDAC5*	nonsynonymous SNV	NM_001015053	p.P667S	1.56%	NA
chr17	42171112	G	A	*HDAC5*	nonsynonymous SNV	NM_001015053	p.A63V	1.56%	NA
chr19	10883235	G	A	*DNM2*	nonsynonymous SNV	NM_001005360	p.D106N	1.56%	Conflicting_interpretations_of_pathogenicity
chr19	10886450	G	A	*DNM2*	nonsynonymous SNV	NM_001005360	p.E153K	1.56%	NA
chr19	10904440	G	A	*DNM2*	nonsynonymous SNV	NM_001005360	p.G346D	1.56%	NA
chr19	10906742	G	A	*DNM2*	nonsynonymous SNV	NM_001005360	p.G401E	1.56%	NA
chr19	10935863	T	A	*DNM2*	nonsynonymous SNV	NM_001005362	p.M671K	1.56%	NA
chr19	10940882	C	-	*DNM2*	frameshift deletion	NM_001005362	p.P787fs	3.13%	NA
chr19	17943384	A	T	*JAK3*	nonsynonymous SNV	NM_000215	p.L875H	1.56%	NA
chr19	17943438	A	G	*JAK3*	nonsynonymous SNV	NM_000215	p.L857P	1.56%	NA
chr19	17945496	G	A	*JAK3*	nonsynonymous SNV	NM_000215	p.P745L	1.56%	NA
chr19	17945918	A	G	*JAK3*	nonsynonymous SNV	NM_000215	p.V674A	3.13%	NA
chr19	17945969	C	T	*JAK3*	nonsynonymous SNV	NM_000215	p.R657Q	1.56%	Likely_pathogenic
chr19	17949108	C	T	*JAK3*	nonsynonymous SNV	NM_000215	p.M511I	1.56%	NA
chr19	54647202	C	T	*CNOT3*	stopgain	NM_014516	p.Q40X	1.56%	NA
chr19	54647241	A	G	*CNOT3*	nonsynonymous SNV	NM_014516	p.K53E	1.56%	NA
chr19	54647429	-	CCATC	*CNOT3*	frameshift insertion	NM_014516	p.S68fs	1.56%	NA
chr19	54649347	T	A	*CNOT3*	nonsynonymous SNV	NM_014516	p.I166N	3.13%	NA
chr19	54649671	T	-	*CNOT3*	frameshift deletion	NM_014516	p.P243fs	3.13%	NA
chr19	54652052	C	T	*CNOT3*	nonsynonymous SNV	NM_014516	p.P355L	1.56%	NA
chr21	36164792	-	GGGT	*RUNX1*	frameshift insertion	NM_001001890	p.T334fs	1.56%	NA
chr21	36164881	C	T	*RUNX1*	nonsynonymous SNV	NM_001001890	p.D305N	3.13%	Uncertain_significance
chr21	36259163	-	GG	*RUNX1*	frameshift insertion	NM_001001890	p.K83fs	1.56%	NA
chr22	30403281	G	-	*MTMR3*	frameshift deletion	NM_021090	p.G284fs	1.56%	NA
chr22	30416297	C	-	*MTMR3*	frameshift deletion	NM_021090	p.S883fs	1.56%	NA
chr22	30416527	A	G	*MTMR3*	nonsynonymous SNV	NM_021090	p.N960S	1.56%	NA
chr22	41545906	A	C	*EP300*	nonsynonymous SNV	NM_001429	p.T841P	1.56%	NA
chr22	41565529	G	A	*EP300*	nonsynonymous SNV	NM_001429	p.D1399N	1.56%	Likely_pathogenic
chr22	41574196	A	G	*EP300*	nonsynonymous SNV	NM_001429	p.M2161V	1.56%	Likely_benign
chrX	15821891	C	T	*ZRSR2*	nonsynonymous SNV	NM_005089	p.A95V	1.56%	NA
chrX	15841013	G	A	*ZRSR2*	nonsynonymous SNV	NM_005089	p.R366K	1.56%	NA
chrX	15841062	C	A	*ZRSR2*	nonsynonymous SNV	NM_005089	p.N382K	1.56%	NA
chrX	39931781	-	A	*BCOR*	frameshift insertion	NM_001123383	p.T940fs	1.56%	NA
chrX	39931966	A	G	*BCOR*	nonsynonymous SNV	NM_001123383	p.V878A	1.56%	Benign
chrX	39932728	G	A	*BCOR*	nonsynonymous SNV	NM_001123383	p.P624L	1.56%	NA
chrX	39933752	C	T	*BCOR*	nonsynonymous SNV	NM_001123383	p.A283T	1.56%	NA
chrX	44928980	G	A	*KDM6A*	nonsynonymous SNV	NM_001291418	p.A615T	1.56%	NA
chrX	44929511	C	T	*KDM6A*	nonsynonymous SNV	NM_001291418	p.L792F	1.56%	NA
chrX	44942750	-	CGTGTTTGTTCT	*KDM6A*	nonframeshift insertion	NM_001291418	p.V1031delinsVRVCS	1.56%	NA
chrX	133527636	C	T	*PHF6*	stopgain	NM_001015877	p.R116X	1.56%	NA
chrX	133547940	C	T	*PHF6*	stopgain	NM_001015877	p.R225X	3.13%	Pathogenic
chrX	133549123	-	CC	*PHF6*	frameshift insertion	NM_001015877	p.L269fs	1.56%	NA
chrX	133549123	-	GA	*PHF6*	frameshift insertion	NM_001015877	p.L269fs	1.56%	NA
chrX	133549136	C	T	*PHF6*	stopgain	NM_001015877	p.R274X	1.56%	Pathogenic
chrX	133549151	G	C	*PHF6*	nonsynonymous SNV	NM_032335	p.V280L	1.56%	NA
chrX	133551269	A	T	*PHF6*	nonsynonymous SNV	NM_001015877	p.H302L	1.56%	NA
chrX	133551319	C	T	*PHF6*	stopgain	NM_001015877	p.R319X	1.56%	NA
chrX	140983151	A	G	*MAGEC3*	nonsynonymous SNV	NM_138702	p.R336G	1.56%	NA
chrX	153629100	A	G	*RPL10*	nonsynonymous SNV	NM_001256580	p.M148V	1.56%	NA
